# Voice-selective prediction alterations in nonclinical voice hearers

**DOI:** 10.1038/s41598-018-32614-9

**Published:** 2018-10-03

**Authors:** Ana P. Pinheiro, Michael Schwartze, Sonja A. Kotz

**Affiliations:** 1 Faculdade de Psicologia, Universidade de Lisboa, Lisboa, Portugal; 2 Neuropsychophysiology Lab, School of Psychology, University of Minho, Braga, Portugal; 3 Faculty of Psychology and Neuroscience, Maastricht University, Maastricht, The Netherlands; 4 Department of Neuropsychology, Max-Planck-Institute for Human Cognitive and Brain Sciences, Leipzig, Germany

## Abstract

Auditory verbal hallucinations (AVH) are a cardinal symptom of psychosis but also occur in 6–13% of the general population. Voice perception is thought to engage an internal forward model that generates predictions, preparing the auditory cortex for upcoming sensory feedback. Impaired processing of sensory feedback in vocalization seems to underlie the experience of AVH in psychosis, but whether this is the case in nonclinical voice hearers remains unclear. The current study used electroencephalography (EEG) to investigate whether and how hallucination predisposition (HP) modulates the internal forward model in response to self-initiated tones and self-voices. Participants varying in HP (based on the Launay-Slade Hallucination Scale) listened to self-generated and externally generated tones or self-voices. HP did not affect responses to self *vs*. externally generated tones. However, HP altered the processing of the self-generated voice: increased HP was associated with increased pre-stimulus alpha power and increased N1 response to the self-generated voice. HP did not affect the P2 response to voices. These findings confirm that both prediction and comparison of predicted and perceived feedback to a self-generated voice are altered in individuals with AVH predisposition. Specific alterations in the processing of self-generated vocalizations may establish a core feature of the psychosis continuum.

## Introduction

Why do some people hear voices in the absence of external stimulation? Self-voice processing relies in part on the capacity to predict the sensory consequences of self-generated vocalizations and on rapidly detecting a discrepancy between predicted and perceived sensations^1,2^. Auditory cortex activity is suppressed in response to self-generated (i.e., fully predictable) compared to external (i.e., less predictable) voices^3–8^. The suppression of sensory cortical responses to self-generated stimuli most likely reflects the tagging of sensations as self-produced to avoid confusion with sensations in the external environment^5,9^. There is compelling evidence that auditory verbal hallucinations (AVH; the perception of voices in the absence of a voice) result from the inability to predict the sensory consequences of self-generated signals due to a dysfunctional internal forward model^10,11^.

## Neural Signatures of the Internal Model

Studies exploring the internal forward model in voice/sound processing have assessed auditory cortex activity by using one of two comparison strategies: listening to self-produced voice in speech production *vs*. listening to pre-recorded self-voice^7,9^ or listening to a sound triggered by a button press *vs*. passively listening to the same sound^12–15^. As the latter paradigm overcomes artifacts related to self-generated voice production (bone conduction; motor activity), it provides some advantages over the monitoring of activity during speech production. Both strategies focus on neural correlates of two aspects of the internal forward model: the *efference copy* (a copy of the motor command that is sent to the auditory cortex via the cerebellum and supports the computation of predicted sensory consequences) and the *corollary discharge* (the expected sensory feedback resulting from a self-generated action)^9,15–17^. Suppression of sensory cortical responses to self-generated stimuli is assumed to reflect the tagging of sensations as self-produced to avoid confusion with sensations originating from the external environment^17^.

Event-related potential (ERP) studies have highlighted the role of the auditory N1 as a putative measure of sensory prediction. The N1 amplitude is suppressed in response to self-generated relative to externally generated voices^4–8^. Similarly, a tone elicited by a button-press leads to an N1 suppression when compared to an externally presented tone^12–15,18,19^. As the auditory N1 is primarily generated in the primary and secondary auditory cortices^20–22^, these findings suggest that the suppression effect associated with the efference copy reflects reduced activity in these brain regions. When auditory feedback does not match the predicted sensation, a prediction error signal is generated, resulting in an increased N1 response^3,15^. Larger N1 responses may signal increased attention to an unexpected sensory event (prediction error)^23–25^.

Sensory-related suppression effects have also been observed in the P2 component of the ERP^3,26^. Whereas N1 suppression is typically directly associated with the expected sensory feedback, the P2 may indicate a more conscious distinction between self-generated and externally-generated sensory events^15,18,19^. However, P2 suppression is not consistently reported. Whereas P2 suppression in response to self-generated tones was observed in button-press tasks (for example^15,18,19^), lack of P2 suppression to self-generated speech sounds in talking tasks has been proposed as a mechanism that allows preservation of the sensory experience of voice feedback during speech generation^27^. These inconsistent findings indicate that the type of task may account for differences in P2 amplitude modulation.

Of note is that these ERP studies focus on neural responses obtained after the onset and during the processing of auditory feedback. However, patterns of neural activity preceding sensory feedback can shed light on the critical stage of sensory prediction formation *per se*. Specifically, pre-stimulus alpha activity is suggested to reflect the prediction of expected sensory consequences of an action^28,29^. Pre-stimulus alpha power is enhanced in sensory cortices prior to self-induced speech^28^, pure tones^29^, or visual stimuli^30^. Further, increased pre-stimulus alpha power for self-generated sounds is associated with larger N1 suppression^29^. Hence, suppression may index the transfer of an efference copy of a motor command to the auditory cortex, whereas the N1 modulation may reflect how well the sensory consequences of an action match or mismatch (reflecting the magnitude of the prediction error), while pre-stimulus oscillatory power may reflect prediction *per se*^29^.

## Internal forward Model and Auditory Verbal Hallucinations

AVH are one of the cardinal symptoms of schizophrenia and experienced by up to 70% of schizophrenia patients^31^. However, they are also present in 6–13% of general population^32,33^. The experience of AVH in psychotic and nonpsychotic individuals seems to engage similar cognitive mechanisms and brain areas^34,35^. This suggests a neural substrate specific to AVH rather than schizophrenia. Nonetheless, despite numerous attempts to explain the neurofunctional mechanisms of AVH, they remain one of the notoriously unexplained symptoms in psychosis.

A substantial body of evidence shows that a failure to distinguish between internally and externally generated sensory signals (e.g., one’s own voice *vs*. somebody else’s voice) may underlie the experience of AVH^34,36,37^. This is reflected in a reduced N1 suppression effect when listening to real-time feedback of one’s own voice^6,11,38^ or in button-press tasks (contrasting self-initiation of a sound with passive exposure to the same sound)^13^. However, the later P2 ERP component as well as pre-stimulus oscillatory EEG activity have not been systematically analyzed in these studies. Further, as most of the existing studies involved chronic schizophrenia patients, it is possible that confounding effects associated with medication, hospitalization, and the presence of negative symptoms may have mediated the reported N1 results.

The study of nonpsychotic individuals who hear voices thus provides a necessary next step to understand the role of altered voice processing in AVH. These individuals are characterized by an increased tendency to falsely report the presence of a voice in bursts of noise^39^ or in conditions of stimulus ambiguity in signal detection tasks^35^. Moreover, they recognize words in degraded speech earlier than controls and before being explicitly informed of its intelligibility^40^. Nonetheless, it remains unclear how these individuals process self-generated *vs*. externally-generated voice and sound feedback. If altered sensory feedback is observed as a function of increased hallucinatory predisposition, this would support the psychosis continuum hypothesis^31^, revealing common physiological brain processes underlying psychotic-like symptoms in nonpsychotic participants.

## The Current Study and Hypotheses

The current study probed whether and how hallucination predisposition modulates the processing of fully predictable (i.e., self-generated) and less predictable (i.e., externally generated) auditory stimuli. By presenting both tones and voices after or in the absence of an action (button press), we examined whether potential problems with prediction formation (pre-stimulus alpha power) and sensory feedback (N1 and P2) are a characteristic in this sample of the general population, and whether the processing of simple (tones) and more complex (voice) auditory stimuli differ in this regard.

A well-established button-press paradigm^15,18,19^ was used. Next to investigating classical ERP components (N1 and P2), we performed a time-frequency analysis of pre-stimulus EEG activity in the alpha range (8–12 Hz). In line with the notion that the efficacy of an internal model is a crucial determinant of the experimental performance, these measures were taken as indices for three different processes: the formation of a prediction or efference copy (pre-stimulus alpha power); the comparison between predicted and perceived sensory feedback (N1); the conscious detection of a self-initiated sound (P2).

Our central hypothesis was that the experience of hallucinated voices involves alterations in the internal forward model. If psychotic-like experiences, such as AVH, are elicited by the same underlying neurocognitive mechanisms as in schizophrenia, nonpsychotic individuals with high hallucination predisposition (HP) should exhibit a similar pattern of EEG activity to that observed in schizophrenia. By forming an efference copy based on finger tapping, the auditory cortex will be prepared for incoming sensation and its activity will be attenuated in participants with lower HP (N1/P2 suppression) but not in those with higher HP^13^. Further, we hypothesized that pre-stimulus alpha power would be reduced for self-generated stimuli as a function of increased HP, reflecting altered prediction formation. We specifically hypothesized that altered prediction and sensory feedback in high HP would be enhanced for voice stimuli^41^.

## Methods

### Participants

The study involved two stages. In stage 1, a large sample of college students from different Universities in Portugal (*N* = 354) was recruited to complete an on-line version of the 16-items Launay-Slade Hallucination Scale (LSHS)^42^. The total score ranges between 0 and 64, with higher scores indicating higher hallucination predisposition. Responses are provided on a 5-point scale (0 = “certainly does not apply to me”; 1 = “possibly does not apply to me”; 2 = “unsure”; 3 = “possibly applies to me”; 4 = “certainly applies to me”). In stage 2, we recruited 49 participants from stage 1, who consented to be contacted for further research on voice processing. Over a 12-month recruitment period, 49 individuals were interviewed in more detail about their experiences and clinical history. All participants completed a thorough clinical assessment that established that for those who reported hearing ‘voices’ (and thus scored higher on the scale), voices were distinct from thoughts, were experienced at least once a month, were unrelated to drug or alcohol abuse, and that participants did not have a psychiatric diagnosis or received a psychiatric diagnosis in relation to voice-hearing.

From the 49 participants recruited, 32 participants varying in their LSHS scores (total score: *M* = 22.72, *SD* = 14.03, range = 0–51; auditory score: *M* = 3.19, *SD* = 2.95, range = 0–9) accepted to partake in an EEG experiment (*M*_*ag*e_ = 22.77, *SD* = 4.06, age rage = 18–32 years; 18 females). Note that recruitment of nonpsychotic voice hearers represents a challenging process due to concerns about stigma as noted in previous studies^40^. *A priori* power calculations using G*Power-3 statistical software^43^ indicated that with a α = 0.05 and power (1-error probability) = 0.90, a sample of a minimum 24 (based on a medium effect size of 0.25) participants would be required to allow the detection of such effects.

Participants were all right-handed^44^, reported normal or corrected-to-normal visual acuity, and normal hearing. All participants provided informed consent and were reimbursed for their time, either by course credits or a voucher. The study was conducted in accordance with the Declaration of Helsinki and was approved by the local Ethics Committee of the University of Minho, Braga (Portugal).

### Stimuli

A 680 Hz tone (50 ms duration; 70 dB sound pressure level [SPL]) and a pre-recorded self-voice speech sound (vowel/a/) were presented in Experiment 1 and 2, respectively. Before Experiment 2, a voice recording session took place: participants were instructed to repeatedly vocalize the syllable “ah”. Recordings were made with an Edirol R-09 recorder and CS-15 cardioid-type stereo microphone^37,45^. After the recording, the best voice sample of the vowel /a/ from each participant (i.e., constant prosody; maximum duration of 300 ms) was selected. The voice sample was edited to eliminate background noise (using Audacity software), and a Praat script was applied to normalize intensity at 70 dB. The stimulus for each participant was saved as.WAV file. Hence, all voice stimuli across participants had the same duration (300 ms) and intensity (70 dB SPL).

### Procedure

**Figure 1:**
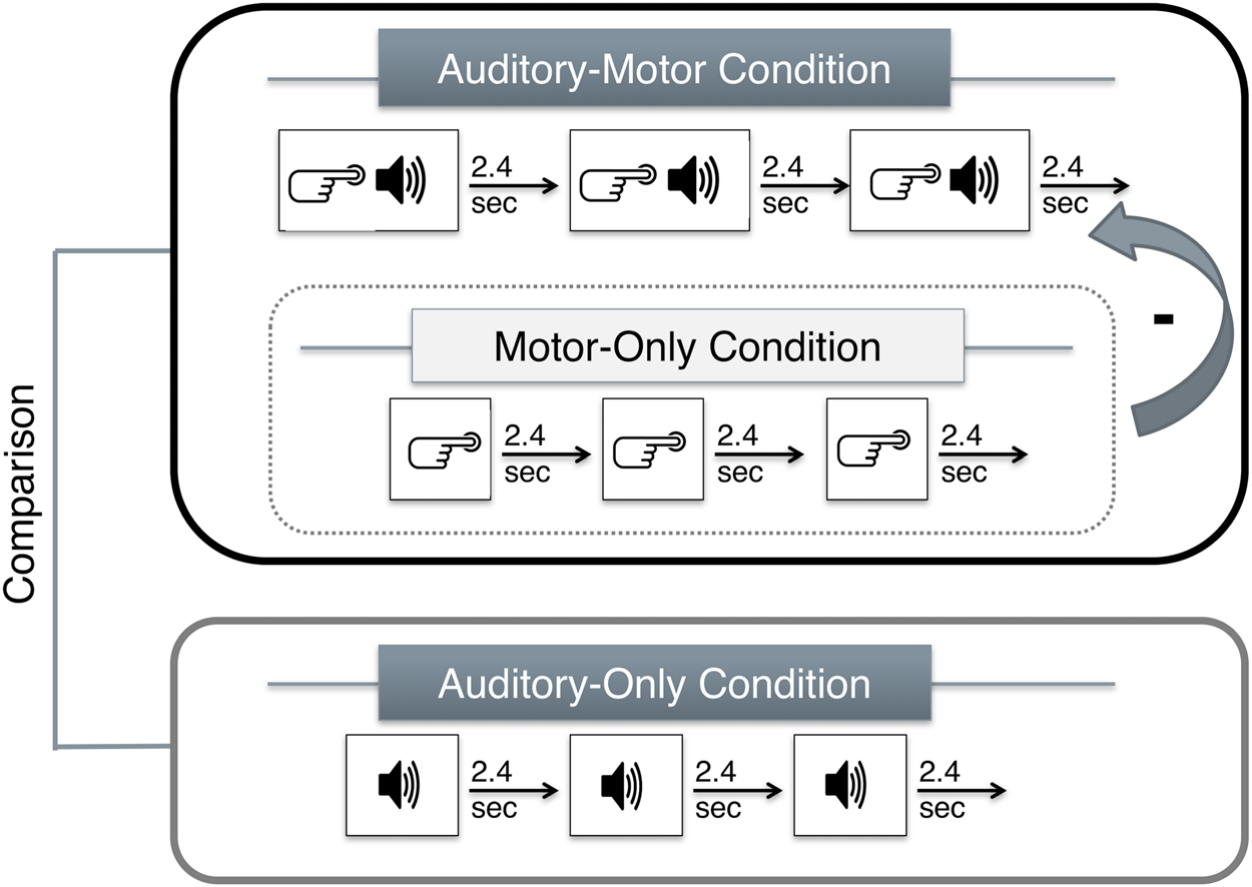
Schematic illustration of the three experimental blocks. A fixation cross was presented in the middle of a computer screen. Participants were instructed to change the index finger after 50 trials to ensure that the motor activation pattern was similar across participants during the AMC and AOC conditions, following indications on the screen (to avoid counting or cognitive demands associated with finger memorization). Change of index fingers was counterbalanced across participants. *Notes:* AMC = auditory-motor condition; AOC = auditory-only condition; MOC = motor-only condition. The AMC was corrected for motor activity based on a difference waveform (AMC-MOC). The statistical analysis involved the comparison of auditory ERPs elicited by the (corrected) AMC and the AOC.

In the EEG experiments, participants sat comfortably at a distance of 100 cm from a desktop computer monitor in a sound-attenuated and electrically shielded room. Each experiment included three conditions (see Fig. 1): auditory-motor (AMC), auditory-only (AOC), and motor-only (MOC)^15,18,19^. In the AMC, a button press instantaneously elicited a tone (Experiment 1) or the prerecorded voice of the participant (Experiment 2). Participants pressed a button approximately every 2.4 seconds. In the AOC, participants were instructed to passively listen and attend to the tones (Experiment 1) or to their pre-recorded self-voice (Experiment 2). The acoustic stimulation from the AMC was recorded on-line and used as the auditory sequence that was passively presented to participants in the AOC. The MOC represents a control condition that allowed controlling for motor-related artifacts via a button press (AMC-MOC): participants performed self-paced button presses approximately every 2.4 seconds but no tone (Experiment 1) or voice (Experiment 2) was elicited by the button press. No measurable sound was emitted by the presses. The AMC always preceded the AOC but the MOC was randomized across participants.

The experimental blocks were preceded by two training blocks^15,18,19^. The training block contained 200 trials. Participants performed correct taps in 75% of trials. No feedback was provided during the experimental blocks. In each of the AMC and AOC, 100 trials were recorded. The MOC in each experiment consisted of 100 trials. Both experiments took place in the same EEG session, but their order was counterbalanced across participants.

The presentation and timing of the stimuli was controlled by Presentation software (version 16.3; Neurobehavioral Systems, Inc.). Auditory stimuli were presented via Sennheiser CX 300-II headphones. A BioSemi tapping device was used to record the finger taps.

### EEG Data Acquisition and Analysis

EEG data were recorded using a 64-channel BioSemi Active Two system in a continuous mode at a digitization rate of 512 Hz and stored on hard disk for later analysis.

**Figure 2:**
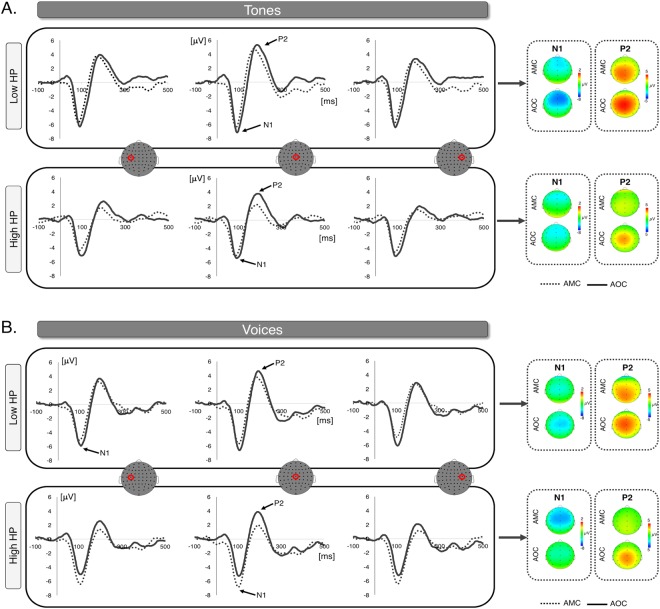
Grand average waveforms contrasting self-triggered and externally triggered tones (Panel A) and self-voices (Panel B) at electrodes C3, Cz and C4. A median split was performed on the data to illustrate differences in the processing of self- and externally triggered stimuli as a function of HP (high HP: ≥26, *N* = 17, range LSHS_Total_: 26–51; low HP: <26, *N* = 15, range LSHS_Total_: 0–25). Topographic maps show voltage distribution in the 80–120 ms (N1) and 175–215 ms (P2) time windows. *Notes:* HP = hallucination predisposition; AMC = auditory-motor condition; AOC = auditory-only condition.

EEG data preprocessing details are presented as Supplementary Material. The ERP analysis followed Knolle *et al*.^15,18,19^. The waveforms revealed two components: a negative component peaking at approximately 100 ms and a positive one peaking at approximately 200 ms (see Fig. 2). Peak amplitudes were calculated in the time windows of 70–110 ms for the N1 and 170–210 ms for the P2^15,18,19^.

In EEG time-frequency analyses, power analysis was performed based on time-frequency magnitude values. The mean power in the alpha band (8–12 Hz) was calculated in a pre-stimulus interval (−250 to 0 ms)^29^.

Amplitude and power data were extracted at four regions of Interest (ROI) comprising: left anterior (F7, F3, FT7, FC3, T7, C3), right anterior (F8, F4, FT8, FC4, T8, C4), left posterior (TP7, CP3, P7, P3, PO7, PO3), and right posterior (TP8, CP4, P8, P4, PO8, PO4) electrode positions^15,18,19^.

### Statistical Data Analyses

Amplitude and power data were analyzed with mixed linear models using the lmer4^46^ and lmerTest^47^ packages in the R environment (R3.4.3. GUI 1.70), which were used to estimate fixed and random coefficients. In contrast to the more traditional repeated-measures ANOVA analysis, LMER allows controlling for the variance associated with random factors such as random effects for participants in ERP amplitude and EEG power measures^48^. The default variance-covariance structure, i.e. the unstructured matrix, was used^49^.

## Results

### Event-related-potentials

#### N1

Intra-class correlation coefficients indicated that 41% of the total variance in the N1 response was accounted for by differences between participants. A Gaussian distribution of residuals was selected to run the mixed model and probability plots (quantile-quantile plots) confirmed its adequacy. Amplitude was included as outcome, participants were included as random effects, whereas stimulus type (tone, voice), condition (self, external), ROI, and LSHS_Total_ were included as fixed effects.

The effects of stimulus type, condition, and ROI were significant. Specifically, we replicated the sensory N1 suppression effect: N1 was more negative for externally-generated compared to self-generated auditory stimuli (β = −0.9498, SE = 0.3934, *t* = −2.414, *p* = 0.016; see Fig. 2). Further, we observed that the N1 response was overall more negative for voices compared to tones (β = −1.031, SE = 0.3934, *t* = −2.621, *p* = 0.009). The typical frontocentral distribution of the N1^50,51^ was confirmed by the observation of less negative amplitude in the left posterior and right posterior ROIs compared to the left anterior ROI (*vs*. left posterior – β = 1.4097, SE = 0.1676, *t* = 8.410, *p* < 0.001; *vs*. right posterior – β = 1.3882, SE = 0.1676, *t* = 8.282, *p* < 0.001), and also to the right anterior ROI (*vs*. left posterior – β = 1.6817, SE = 0.1653, *t* = 10.172, *p* < 0.001; *vs*. right posterior – β = 1.6602, SE = 0.1653, *t* = 10.042, *p* < 0.001). The model identified a positive effect of LSHS_Total_ scores on the N1 amplitude, indicating that differences in hallucination predisposition accounted for N1 amplitude modulations (β = 0.0361, SE = 0.0159, *t* = −2.621, *p* = 0.009; see Fig. 2).

**Figure 3:**
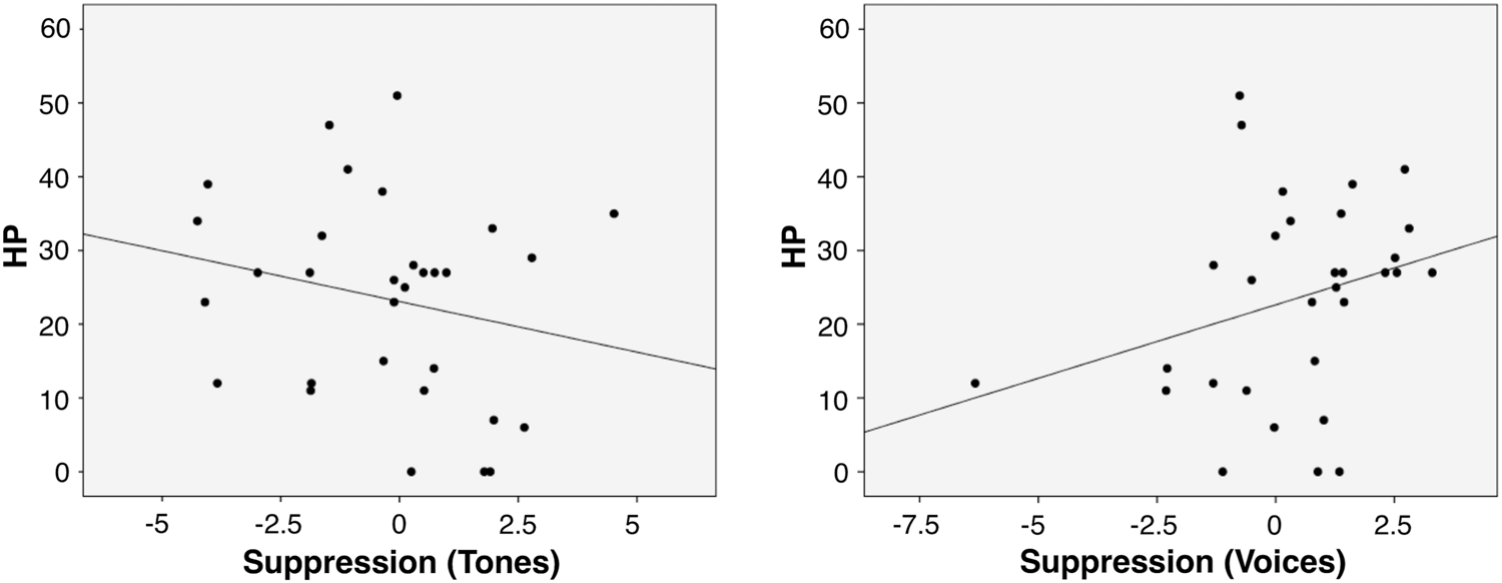
Relationship between hallucination predisposition (LSHS_Total_) and N1 suppression to tones and voices.Note. Values represent difference in N1 amplitude between AOC and AMC conditions (negative values indicate less negative N1 in the AMC compared to AOC [AMC<AOC]; positive values indicate more negative N1 in the AMC compared to AOC [AMC>AOC].

Based on our hypotheses, we tested whether LSHS_Total_ scores had a specific impact on the N1 amplitude as a function of stimulus type and condition, probing the interaction between the three predictors (see Table 1). For the sake of simplicity and facility in the interpretation, we defined the variable “interaction” (B) with 4 levels (2 stimulus types × 2 conditions) and tested the following model in R: m.N1 <− lmer(N1 ~ LSHS_Total * B + ROI  + (1|Subject), data = N1, REML = FALSE); summary(m.N1), in which N1$B <− interaction(N1$StimulusType, N1$Condition). An increase in LSHS_Total_ scores was associated with a less negative N1 response for self-generated tones compared to self-generated voices (β = 0.0423, SE = 0.0120, *t* = 3.535, *p* < 0.001) as well as in a less negative N1 for externally-generated voices compared to self-generated voices (β = 0.0301, SE = 0.0120, *t* = 2.517, *p* = 0.012; see Fig. 3). Specifically, the N1 amplitude in response to the self-generated voice was expected to be more negative than the N1 amplitude for the externally-generated voice if LSHS_Total_ > 18.94, and the N1 amplitude for the self-generated voice was expected to be more negative than the amplitude for the self-generated tone if LSHS_Total_ > 15.83. A separate analysis of midline electrodes (considering anterior [Fz, Cz] vs. posterior [CPz, Pz] electrode sites) using the same statistical model described before revealed similar effects.

**Table 1:** Linear mixed effects model of N1 amplitude including the effect of hallucination predisposition.

Variable	Estimate	SE	t-value	Pr(>|z|)
**Fixed Effects**				
Intercept	−5.44527	0.48904	−11.135	<0.001***
LSHS_Total_	0.04619	0.01800	2.566	0.013612*
LSHS_Total_* Self* Tone (vs. External Tone)	0.02261	0.011198	1.888	0.0596
LSHS_Total_* External* Voice (vs. Self-voice)	0.030137	0.011976	2.517	0.012177
LSHS_Total_* Self* Tone (vs. Self-Voice)	0.042339	0.011976	3.535	<0.001***
LSHS_Total_* External* Voice (vs. External Tone)	0.01041	0.01198	0.869	0.3852
**Groups**	**Name**	**Variance**	**SD**	
**Random Effects**				
Subject	(Intercept)	1.538	1.240	
Residual		1.749	1.323	

*Notes*. SE = standard error; SD = standard deviation; *p < 0.05; **p < 0.01; ***p < 0.001. Degrees of freedom for Fixed Effects: df = 480.00 (except Intercept: df = 50.01).

To probe the specific effect of AVH, we ran the same model replacing LSHS_Total_ with LSHS_Auditory_ (sum of items 4 – “In the past, I have had the experience of hearing a person’s voice and then found no one was there”, 8 – “I often hear a voice speaking my thoughts aloud”, and 9 – “I have been troubled by voices in my head”) as a fixed factor. The effect of LSHS_Auditory_ on the N1 amplitude was significant (β = 0.1840, SE = 0.0849, *t* = 2.167, *p* = 0.0352). Further, the interaction of stimulus type and condition revealed a specific effect of the N1 amplitude modulation for the self-generated voice (β = −0.1242, SE = 0.0600, *t* = −2.069, *p* = 0.039).

#### P2

The analysis of P2 amplitude followed the same procedure as described above. Removing LSHS_Total_ increased the goodness of fit of the model (χ^2^(10) = 2.859, *p* < 0.001), even though the Akaike’s Information Criterion (AIC^52^) for the complete model (AIC = 1849.9) was slightly lower than the AIC for the model without LSHS_Total_ (AIC = 1850.8). Hence, LSHS_Total_ was not included as a predictor of the P2 amplitude modulation.

The model showed that self-generated auditory stimuli were associated with a less positive P2 amplitude compared to externally-generated auditory stimuli (β = 0.4626, SE = 0.1670, *t* = 2.770, *p* = 0.0058; see Fig. 2). Stimulus type did not significantly predict the P2 amplitude modulation (β = −0.2332, SE = 0.1670, *t* = −1.397, *p* = 0.1631). A separate analysis of midline electrodes (considering anterior [Fz, Cz] vs. posterior [CPz, Pz] electrode sites) using the same statistical model described before revealed similar effects.

### Pre-stimulus alpha power

**Figure 4:**
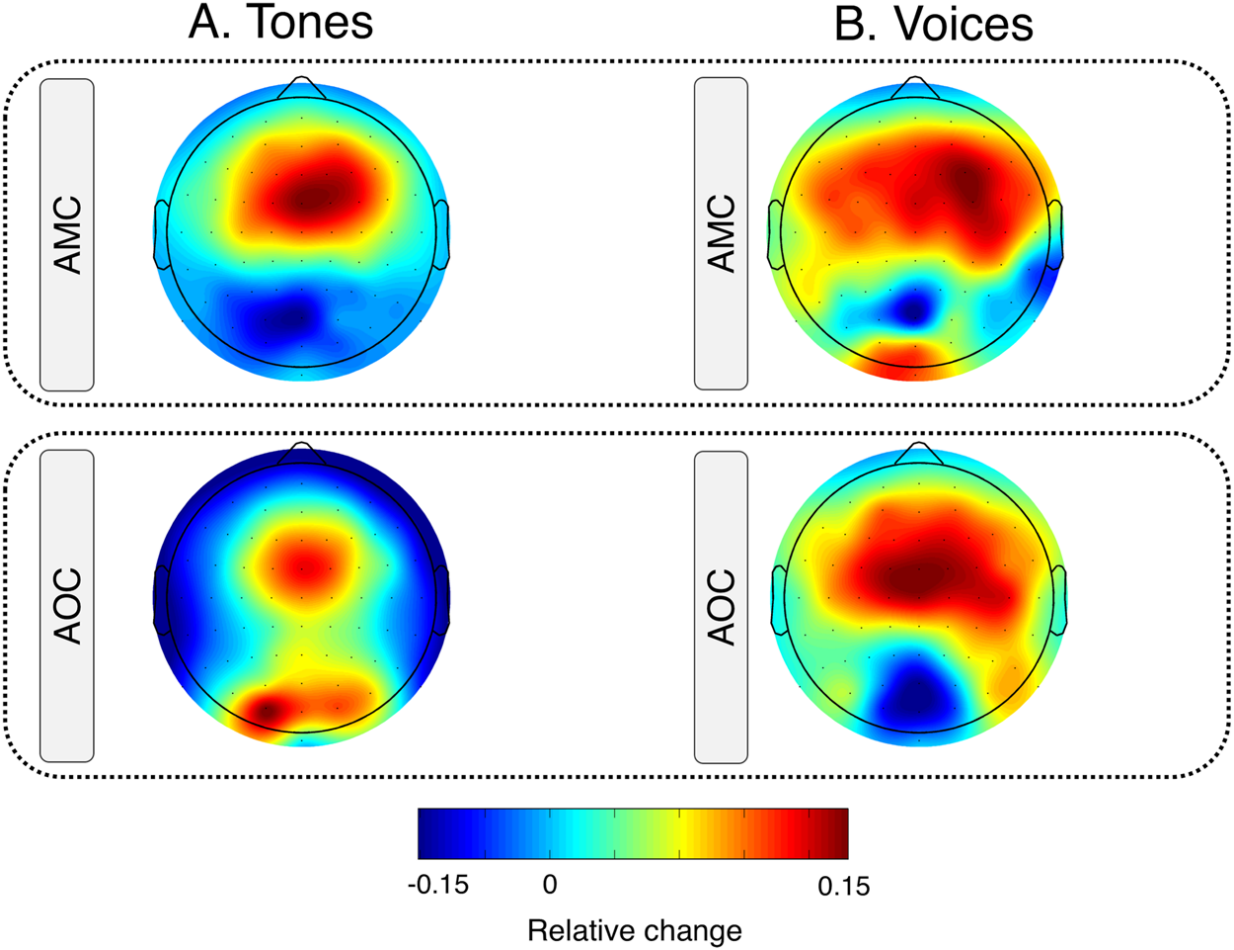
Maps showing the topographical distribution of pre-stimulus alpha power for the different conditions and stimulus types. Values are averaged across the 8–12 Hz frequency band in a time window of 250 ms before stimulus onset.

The model including LSHS as a predictor had the best goodness of fit (χ^2^(13) = 36.957, *p* < 0.001; AIC with LSHS_Total_ = −1691, AIC without LSHS_Total_ = −1662; see Table 2). Alpha power was decreased before sounds that were externally-generated compared to self-generated sounds (β = −0.0115, SE = 0.0054, *t* = −2.121, *p* = 0.035; see Table 2 and Fig. 4).

**Table 2:** Linear mixed effects model of pre-stimulus alpha power including the effect of hallucination predisposition.

Variable	Estimate	SE	t-value	Pr(>|z|)
**Fixed Effects**				
Intercept	0.02331	0.01452	1.606	0.114183
LSHS_Total_	0.00229	0.0005372	3.810	<0.001***
LSHS_Total_* Self* Tone (vs. External Tone)	0.0006327	0.0003783	1.673	0.095054
LSHS_Total_ * External * Voice (vs. Self-Voice)	−0.001799	0.0003783	−4.756	<0.001***
LSHS_Total_* Self* Tone (vs. Self-Voice)	−0.001464	0.0003783	−3.870	<0.001***
LSHS* External* Tone (vs. External Voice)	0.0002974	0.0003783	0.786	0.432177
**Groups**	**Name**	**Variance**	**SD**	
**Random Effects**				
Subject	(Intercept)	0.001294	0.03597	
Residual		0.001745	0.04177	

*Notes*. SE = standard error; SD = standard deviation; *p < 0.05; **p < 0.01; ***p < 0.001. Degrees of freedom for Fixed Effects: df = 480.00 (except Intercept: df = 53.44).

HP specifically influenced alpha activity preceding the self-generated voice (see Table 2). An increase in LSHS_Total_ resulted in a decrease in alpha power preceding self-generated tones compared to self-generated voices (β = −0.0015, SE = 0.0003, *t* = −3.870, *p* < 0.001) and preceding externally triggered voices compared to self-triggered voices (β = 0.0017, SE = 0.0004, *t* = −4.756, *p* < 0.001).

As for the N1, we probed the specific effect of AVH by replacing LSHS_Total_ with LSHS_Auditory_ as a fixed factor. The effect of LSHS_Auditory_ on pre-stimulus alpha power was not significant (β = 0.0015, SE = 0.0023, *t* = 0.623, *p* = 0.538).

## Discussion

Why some people hear voices in the absence of external stimulation remains to be clarified. The current study probed how HP modulates the processing of auditory stimuli (tones *vs*. voices) that are anticipated as a consequence of a motor act (a button press) engaging an internal forward model. The study of nonpsychotic individuals with high HP represents an important step forward in probing the continuum hypothesis of psychosis. Here, we disentangled whether increased HP affects the formation of a prediction or efference copy (pre-stimulus alpha power), the comparison between predicted and perceived sensory feedback (N1), and/or the conscious detection of a self-initiated sound (P2). Our results suggest that an increase in HP is specifically related to alterations both in the generation of an efference copy and in the comparison between predicted and perceived sensory feedback. Further, they show that these alterations are more pronounced during the perception of one’s own voice compared to simple tones.

### The N1 suppression effect is reversed in high HP

Our findings replicate the classical sensory suppression effect (for example^5–12,15,18,19,53^) both for tones and voices: self-generated sounds elicited a smaller N1 compared to externally generated sounds irrespective of stimulus complexity, an effect that was more prominent at anterior electrode sites^50,51^. As the sensory consequences of self-initiated sounds are precisely predicted such that the auditory cortex is prepared to receive sensory feedback, the N1 amplitude is suppressed. We also observed that self-voices elicited larger N1 amplitudes than tones, an effect that might be accounted for by differences in the duration of the two sound categories (50 ms for tones; 300 ms for voices): N1 amplitude was found to decrease with a shorter stimulus duration^54^, and to increase linearly with a longer tone duration^55^. However, given the complexity of factors known to modulate the N1 amplitude, other factors may have accounted for this effect (e.g., attention^54^).

Whereas the N1 attenuation effect to self-initiated tones was not affected by HP, the response to self-generated voices was. Higher HP resulted in an N1 enhancement rather than suppression: self-generated voices elicited a larger N1 amplitude modulation than externally-generated voices. Typically, an increased N1 response to self-generated auditory stimuli has been related to an increase in prediction error. When sensory feedback mismatches a prediction, the N1 suppression effect is reduced^3,56,57^. Further, when predictions are less specific, the N1 suppression effect is smaller^58^. Based on this evidence, one could also argue that when voices are less clearly predicted, sensory feedback is not attenuated. Hence, the increased N1 response to self-triggered voices may indicate that the self-voice is less accurately predicted even when participants with higher HP produce sensory feedback to their voices. Specific predictions are even more relevant in the case of more complex auditory stimuli, for which features such as stimulus frequency, onset, and intensity need to be incorporated into the prediction of the self-voice. Thus, a prediction error may arise from the comparison of a less specific/accurate prediction with the available sensory feedback. A less specific prediction may imply altered self-monitoring of speech, which has been consistently associated with AVH^34,36,37,59–62^.

We cannot rule out the contribution of attentional processes when looking at the enhanced N1 response as a function of increased HP. Increased attention to the to-be-presented voice may have prevented the motor-induced attenuation effect. Cognitive theories of AVH contemplate the role of biased attentional processes in some forms of AVH^63^. Attention and prediction processes may have opposite effects with regards to the N1 attenuation: whereas prediction reduces the N1, attention results in increased N1 amplitude^64,65^. An increased N1 response to self-triggered voices due to increased HP may indicate that participants with high HP focus their attention more on self-voice stimuli^64^.

### The conscious detection of a self-initiated voice is not affected by HP

The P2 response was also suppressed to both self-initiated tones and voices compared to their passive presentation. The existing evidence is less consistent regarding the effects of predictable sensory feedback on the P2. The pattern we found here is compatible with our previous results on tones^15,18,19^. The P2 suppression effect may reflect the conscious detection of sensory feedback as self-generated, playing a role in the distinction of self and externally-produced sounds^15,18,19^. Both groups showed a similar P2 suppression effect for tones and voices. This similarity suggests that the conscious detection of a sound that follows a button-press as self-produced is not affected by HP. This may underlie the preserved sensory experience of a self-generated sound despite reduced sensory suppression. Similar findings (i.e., lack of differences in P2 amplitude relative to controls) were observed in talking or button-press paradigms in schizophrenia^13^ or cerebellar patients^18,19^.

### Increased expectancy for voices in hallucination predisposition

We also observed that HP modulated EEG activity before sound onset. Specifically, HP affected pre-stimulus alpha activity for self-generated voices only: increased HP resulted in increased alpha power. Activity preceding an action (e.g., pressing a button that elicits a sound) has been proposed to reflect the formation of an efference copy^27^. Increased pre-stimulus alpha power has been related to prediction effects (occurring in the medial prefrontal cortex^28^) that allow preparing the auditory cortex for the processing of self-generated sounds^28,29^. Specifically, alpha power is sensitive to the precision of a prediction regarding an upcoming stimulus. As HP participants did not show the expected sensory attenuation effect to self-triggered voices, it is possible that enhanced predictability to relevant features of the self-voice results in increased synaptic gain that reverses the suppressive effects of the prediction (i.e., the N1 to self-triggered voices fails to be suppressed).

### Implications for models of AVH and of a psychosis continuum

Although high HP participants showed a sensory attenuation effect that differs from that previously reported in schizophrenia (lack of a suppression effect)^11^, the increased N1 response to self-voices is partially consistent with an altered internal forward model in AVH. Whereas HP did not alter the processing of externally generated sounds (no difference between external tone and external voice), it did affect self-voice processing. Further, in both ERP and EEG oscillatory activity, the effects of HP were selective for voices, but not tones. A voice-specific rather than generalized, altered sensory prediction may explain why individuals who experience AVH hallucinated voices display the most common abnormal perceptual experience.

It is worth noting that even though this was not explicitly reported in talking paradigms (i.e., in which voice stimuli were tested) in schizophrenia patients with AVH, the inspection of grand average waveforms suggests that HP affected the processing of self-generated vocal sounds more (N1 amplitude was more negative in the talking condition in patients with hallucinations compared to controls) than the processing of externally generated voices (no group differences)^11^. It is possible that along the psychosis continuum, altered sensory feedback that can be predicted as a function of one’s own action, occurs first for auditory stimuli with increased social relevance (e.g., voices) and in clinical stages of the continuum it generalizes to other types of sounds (e.g., simple tones). This hypothesis is admittedly speculative and needs to be tested in future studies with larger samples.

Together, the current findings suggest that sensory feedback to self-voice is altered in people with an AHV predisposition. Specific alterations in the processing of self-generated vocal sounds may thus establish a core feature on the psychosis continuum.

## Electronic supplementary material

Supplementary Material

## Data Availability

The datasets generated during and/or analyzed during the current study are available from the corresponding author on reasonable request.
