# Supplementary material for: Voice-selective prediction alterations in nonclinical voice hearers
Source: Sci Rep. 2018 Oct 3;8:14717. doi: 10.1038/s41598-018-32614-9 (PMC6170384; doi:10.1038/s41598-018-32614-9)
Supplement: Supplementary file 1 — Supplementary Material [file 41598_2018_32614_MOESM1_ESM.doc]

**Voice-selective prediction alterations in nonclinical voice hearers**

Ana P. Pinheiro, Michael Schwartze, & Sonja A. Kotz

**Supplementary Material**

**1. ERP analysis.**

Using EEGLAB 13.1.1b software and in-house developed Matlab functions (The Mathworks), EEG data were band-pass filtered at 0.1-30 Hz filter (1601 Hamming windowed filter) and referenced offline to the average of the left and right mastoids. EEG was baseline corrected using a -100 to 0 ms pre-stimulus interval. Tapping intervals shorter than 1.8 sec or longer than 3.0 sec were treated as errors and excluded from further EEG analyses. Segments were screened for eye movements, muscle artifacts, electrode drifting, and amplifier blocking. The vertical EOG was derived by subtracting the activity measured at an electrode positioned below the left eye from an electrode positioned above it. The horizontal EOG was derived by subtracting the activity measured in electrodes placed at the outer canthi of the eyes. EEG epochs with amplitudes exceeding +/-100 microvolts were rejected. After artifact rejection, at least 75% of the segments per condition per participant entered the analyses. Conditions did not differ in the number of non-rejected epochs (*p*>.05). Grand average waveforms were generated for each condition in each group for both experiments. Motor activity was controlled by computing difference waveforms between the AMC and MOC.

**2. EEG Time-Frequency Analysis**

For time-frequency analyses, EEG data were re-referenced offline to the average reference. Time frequency decomposition was performed using a Complex Morlet’s wavelet transform, applied in 0.25 Hz steps from 4 to 60 Hz at each time point to yield time-frequency (TF) maps of induced power. The constant ratio of central frequency (*c=f0/σf*) was 7 and the multiplication factor (*m*) was 4.^44^ A bandwidth parameter (*F_B_*) was defined as
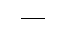
, where *nc* is the number of cycles (considered *nc*=4.46) and *fo* the central frequency of the wavelet. A normalization factor was applied:
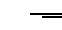
. Baseline activity (-700 to -300 ms) was subtracted from each TF map. The analysis of the phase-locked activity was performed based on time-frequency magnitude values. Pre-stimulus power analysis was conducted with basis on the following equation:


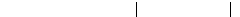


In the equation, *w(t,f)* is the Complex Morlet’s Wavelet and *s_E_(t)* is the evoked response signal (averaged across trials). The mean power in the alpha band (8-12 Hz) was calculated in a pre-stimulus interval (-250 to 0 ms).
